# Oxygen-vacancy-induced half-metallicity and enhanced ferromagnetism in Sr_2_NiMnO_6_: a DFT and Monte Carlo approach

**DOI:** 10.1039/d6ra07202j

**Published:** 2026-10-19

**Authors:** Rifat S. Apu, Mohammad Kamrul Hasan, Anas Marsaad, Md. Harunur Rashid, Rubaiyet I. Haque

**Affiliations:** a Department of Electrical Engineering and Automation, Aalto University Finland; b Department of Mathematics and Physics, North South University Dhaka Bangladesh rubaiyet.haque@northsouth.edu harunur.rashid01@northsouth.edu; c Department of Electrical and Computer Engineering, North South University Dhaka Bangladesh

## Abstract

Realizing room-temperature spintronic functionality requires materials that maintain complete spin polarization at the Fermi level and robust ferromagnetic stability against thermal fluctuations. Here, Sr_2_NiMnO_6_ (SNMO) is introduced as a novel, high-performance double-perovskite candidate, and a mechanism to unlock its half-metallic potential *via* oxygen-vacancy (V_O_) engineering is uncovered. Using a combined DFT, lattice dynamics, and Monte Carlo approach, it is revealed that V_O_ induction triggers a symmetry reduction (from *I*4/*m* to P1), driving a Ni-centered redox transition from a formal Ni^4+^ toward a Ni^2+^-like character, thereby evolving the electronic state from a spin-asymmetric semiconductor to a half-metal. The transition is further characterized by a substantial increase in the carrier effective mass (up to 1.235 *m*_0_), consistent with the emergence of localized polaronic character supporting the material's high-temperature stability and spin-selective transport. This structural-electronic coupling reconfigures the magnetic interaction from a weak superexchange-dominated regime toward an enhanced carrier-mediated exchange, consistent with, but not directly demonstrated by, the localized in-gap Ni states reported here, elevating the saturated magnetic moment to 6.00 *µ*_B_ (from −2.97 *µ*_B_) and the Curie temperature to 480 K. These analyses establish a direct relationship between oxygen-vacancy formation, structural distortion, electronic reconstruction, and magnetic ordering in SNMO, highlighting oxygen-vacancy engineering as a strategy for tuning the spin-dependent properties of double perovskites for high-temperature spintronic applications.

## Abstract 2

Oxygen vacancies drive lattice distortion and Ni redox reconstruction, converting Sr_2_NiMnO_6_ to a half-metal. Enhanced carrier exchange and localized Ni states boost ferromagnetism, enabling robust high-temperature spintronics.

## Graphical abstract

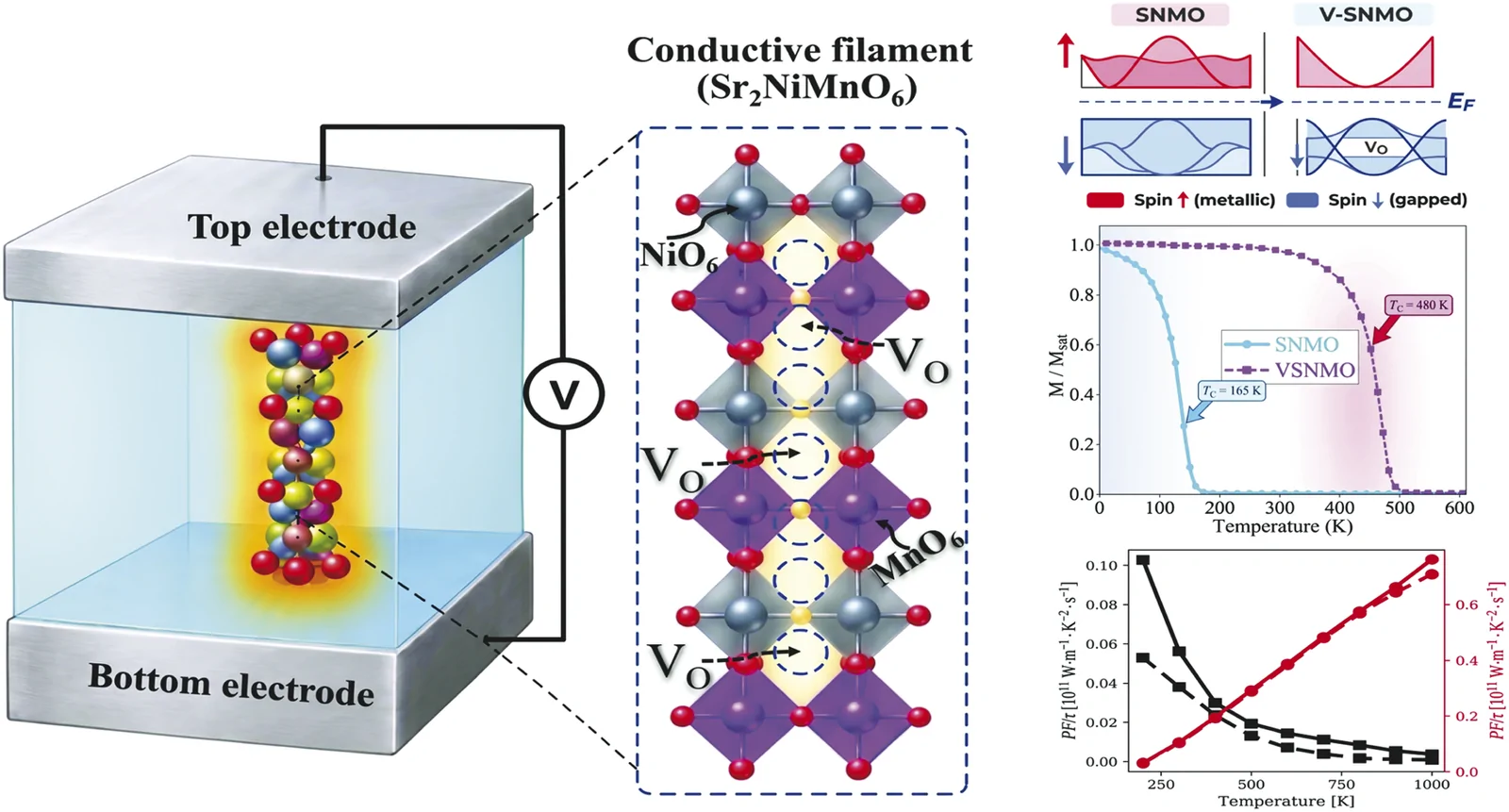

## 1 Introduction

Recent computational studies of related double perovskite oxides report half-metallic ferromagnetism and thermoelectric performance of direct relevance to spintronic applications.^1^ The precise control of spin-polarized electronic transport is a fundamental requirement for the development of next-generation spintronic devices, such as magnetic tunnel junctions and spin-transfer torque memories.^2,3^ Within this context, double perovskite oxides (A_2_BB′O_6_) have emerged as a premier platform due to their structural versatility and the ability to precisely tune exchange interactions through B-site ordering.^4,5^ The archetypal Sr_2_FeMoO_6_ demonstrated that the rock-salt ordering of dissimilar transition-metal cations could generate a fully spin-polarized conduction channel with tunneling magnetoresistance persisting well above room temperature.^6,7^ This motivates the theoretical exploration of new Ni/Mn-based compositions to further optimize these properties; specifically, Sr_2_NiMnO_6_ (SNMO) is proposed as a promising novel material that could host the interplay between localized magnetic moments and itinerant carriers for the purpose of engineering spin-asymmetric electronic states.

In Ni/Mn-ordered double perovskites, the magnetic ground state is typically understood to be governed by a cooperative synergy between two distinct exchange pathways, the t_2g_-O 2p-t_2g_ superexchange (SE) and e_g_-O 2p-e_g_ double-exchange (DE).^8–10^ Although the SE channel provides a robust ferromagnetic backbone, the DE channel is driven by carrier hopping through the hybridized Ni–O–Mn states and is primarily responsible for the itinerant transport properties.^11–13^ However, many of these systems are observed to behave as quasi-half-semiconductors, where both spin channels possess an energy gap, albeit with significant asymmetry.^14,15^ This inherent semiconducting nature often limits electrical conductivity and spin-injection efficiency, creating a fundamental bottleneck for the integration of such materials into high-speed, current-driven spintronic architectures.

The electronic properties of perovskite oxides are known to be exceptionally sensitive to oxygen stoichiometry. Oxygen vacancies (V_O_) are ubiquitous in oxide thin films and are often regarded as parasitic defects that can suppress saturation magnetization.^16,17^ Nevertheless, from a quantum-mechanical perspective, V_O_ can act as a potent double-donor, injecting electrons into the transition-metal 3d manifold and altering the local oxidation states.^17^ Such vacancy-induced metallization has been documented in non-magnetic perovskites like SrTiO_3_,^18^ while in other magnetic oxides, the redistribution of charge has been shown to modulate the spin polarization of the conduction channel.^19^ This raises a critical unresolved question: whether the removal of an oxygen bridge, the very mediator of the SE and DE interactions, destroys the ferromagnetic coupling, or if it can be utilized as a strategic “tuning knob” to drive an electronic phase transition.^20^

While first-principles calculations provide a rigorous description of the electronic ground state at zero temperature, the practical utility of any spintronic material is fundamentally governed by its stability against thermal fluctuations.^11,21^ In ferromagnetic oxides, the transition from a spin-polarized state to a paramagnetic phase is driven by the excitation of magnons, which progressively diminish the net magnetization as the temperature approaches the Curie point (*T*_C_).^21,22^ Consequently, the prediction of a favorable electronic structure at 0 K is necessary, but the material must maintain its magnetic order and spin polarization at or above room temperature to be technologically viable.^22,23^ To bridge this gap between static quantum-mechanical predictions and macroscopic thermal behavior, it is essential to employ statistical mechanical frameworks, such as Monte Carlo simulations, using exchange integrals derived from first-principles calculations.^23^ This DFT + Monte Carlo approach has recently proven reliable for predicting Curie temperatures in half-metallic double perovskites,^15,24,25^ and is especially relevant for defect-engineered systems, since vacancy formation itself is magnetic-state-dependent and modifies exchange coupling strength,^26,27^ as similarly shown for antisite/vacancy disordered double perovskites targeted for spintronic memory.^13,28^

Defect and dopant engineering has also been used to control half-metallicity and magnetism in two-dimensional materials, where ferroelectric polarization switches a metal to a half-metal transition in semi-hydrogenated graphene;^29^ vacancies and transition-metal substitution generate magnetic order in germanane;^30^ and substitutional doping tunes monolayer PtS_2_ between semiconducting, half-metallic and ferromagnetic states.^31^ These platforms rely on adsorption, substitution or substrate control in low dimensions, whereas in the bulk ordered double perovskite studied here, a single-point-defect class, the oxygen vacancy injects electrons directly into the transition-metal manifold and reorganizes the competing exchange network of the host itself.

In this work, the integrated framework is employed to uncover the transformative effect of oxygen-vacancy engineering on the electronic and magnetic landscapes of Sr_2_NiMnO_6_ (SNMO). Combining high-fidelity first-principles calculations with Monte Carlo simulations, it is demonstrated that the introduction of V_O_ triggers a fundamental electronic phase transition from a quasi-half-semiconducting state to a half-metallic phase. It is revealed that this transition is driven by a predominantly Ni-centered reduction from formal Ni^4+^ toward Ni^2+^-like character, which pins an intense in-gap resonance at the Fermi level while concurrently reinforcing the exchange splitting of the minority-spin channel. This mechanism induces a profound spin-selective bifurcation in the transport response, as characterized by the spin-resolved Seebeck coefficient and the electronic figure of merit. These findings establish oxygen-vacancy engineering as a strategy for defect-tunable spintronic materials, where field-driven vacancy migration enables non-volatile memory and spin-based logic by integrating resistive switching and spin-selective transport within a single material platform.

## 2 Methodology

All first-principles calculations were performed using the Vienna *Ab Initio* Simulation Package (VASP),^32^ employing the PAW method within DFT.^33^ The exchange–correlation effects were treated using the GGA,^34^ with the PBE functional.^35^ To accurately capture the strong on-site Coulomb correlations inherent to the localized Ni 3d and Mn 3d manifolds, a rotationally invariant DFT+U approach was implemented.^36^ Hubbard parameters of *U*_eff_ = 5 eV for Ni and 4 eV for Mn were adopted,^37^ consistent with previously reported values for similar double perovskite oxides, to accurately capture the strong on-site Coulomb correlations. The choice of these parameters was further validated by performing a convergence test, ensuring that the electronic band gap and the ferromagnetic ground state remain robust against small variations in *U* (±0.5 eV).^38^

The plane-wave basis set was expanded with a kinetic-energy cutoff of 550 eV. Brillouin-zone integration was performed using a *Γ*-centered Monkhorst–Pack *k*-mesh of (7 × 7 × 5) for structural relaxations and a denser (12 × 12 × 10) mesh for the evaluation of the electronic density of states (DOS) and transport coefficients.^39^ Structural convergence was defined by a total-energy threshold of 10^−6^ eV and residual Hellmann–Feynman forces below 0.01 eV Å^−1^.^40^ Electronic charge distribution and charge redistribution were analyzed using Bader partitioning. Formal oxidation states were assigned through charge-neutral electron counting, while Bader-charge differences were used to identify vacancy-induced electron redistribution.

Oxygen vacancies (V-SNMO) were introduced by removing six oxygen atoms from a 2 × 2 × 1 supercell of the tetragonal *I*4/*m* cell (*Z* = 2, 20 atoms per unit cell), giving a pristine supercell of 80 atoms (48 O sites, stoichiometry Sr_2_NiMnO_6_) and a defective supercell of 74 atoms (42 O sites, stoichiometry Sr_2_NiMnO_5_._25_, *δ* = 0.75 per formula unit), corresponding to a vacancy concentration, expressed as the fraction of oxygen sites removed, of 6/48 = 0.125 = 12.5%, consistent with the originally reported value charge neutrality at this composition reduces the average transition-metal valence by 1.5 per formula unit relative to formal Ni^4+^ and Mn^4+^, placing the system 75% of the way toward the fully reduced Ni^2+^/Mn^4+^ end member Sr_2_NiMnO_5_,^41^ followed by full ionic and cell relaxation, which resulted in a symmetry lowering from the tetragonal *I*4/*m* to the triclinic *P*1 space group,^42^ with a nearest-neighbor vacancy–vacancy distance within the ordering pattern of the six vacancies of ≈3.97 Å. Oxygen vacancy migration barriers were subsequently computed using the climbing-image nudged elastic band (CI-NEB) method^42^ with five intermediate images, relaxed until perpendicular forces fell below 0.02 eV Å^−1^.^43^

Dynamic stability was verified from phonon dispersion calculations using the finite-displacement method implemented in Phonopy,^44^ with second-order force constants obtained from VASP calculations on a 2 × 2 × 2 supercell.^44^ Spin-resolved thermoelectric transport properties, including the Seebeck coefficient, electrical conductivity, electronic thermal conductivity, and power factor, were calculated using semi-classical Boltzmann transport theory within the constant relaxation-time approximation as implemented in BoltzTraP2.^45^ Calculations were performed over 200–1000 K using a dense (16 × 16 × 12) *k*-point mesh to ensure convergence of the transport integrals.

To evaluate the thermal stability of the ferromagnetic state and to determine the Curie temperature (*T*_C_), Monte Carlo simulations were performed based on a classical two-sublattice Heisenberg Hamiltonian,^46^
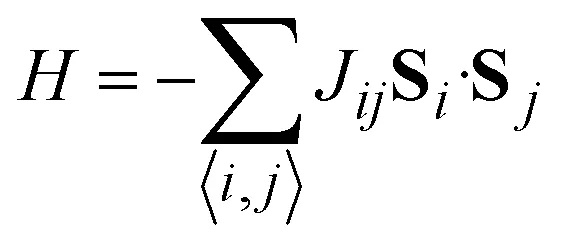
where **S**_*i*_ and **S**_*j*_ represent the unit spin vectors of the Ni and Mn sublattices, and *J*_*ij*_ is the effective exchange interaction between nearest neighbors. The exchange constant *J* was derived from the total energy difference (Δ*E* = *E*_FM_ − *E*_FIM_) obtained from DFT calculations for the tetragonal phase. This is justified by PDOS/Bader results. This total-energy difference is mapped onto the classical Heisenberg Hamiltonian mentioned above *via*
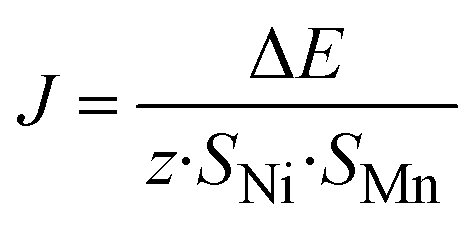
, where *z* = 6 is the number of nearest-neighbor Ni–Mn pairs per formula unit in the rock-salt B-site ordering (three Ni–Mn bonds per formula unit, each flipped from parallel to antiparallel at a cost of 2*J S*_Ni_·*S*_Mn_), *S*_Ni_ = 1 and *S*_Mn_ = 3/2 are the classical spin magnitudes, and Δ*E* is the ferrimagnetic minus ferromagnetic total-energy difference.^25^ This yields *J* = 19.0 meV for the vacancy-engineered phase and *J* = 6.5 meV for the pristine phase. The t_2g_ superexchange network remains largely unchanged between pristine and V-SNMO, while the e_g_ double-exchange channel is the only pathway modified by vacancy-induced carrier injection. Thus, *J* extracted here predominantly tracks the DE contribution in V-SNMO and acts as a composite effective constant in the pristine phase.^15,24,25^

Simulations were carried out on a periodic two-sublattice network of 4096 spins (2048 Ni and 2048 Mn) constructed from the B-site sublattices of the 80-atom pristine and 74-atom defective supercells, using single spin flip Metropolis updates. The spin magnitudes were set to *S*_Ni_ = 1.0 and *S*_Mn_ = 1.5, corresponding to the reduced Ni^2+^ like state and to Mn^4+^, used as fixed classical model spin lengths in both phases. At each temperature, 10^4^ Monte Carlo sweeps were used for thermalization, followed by 10^4^ sweeps for averaging. *T*_C_ was identified as the temperature corresponding to the peak in the magnetic susceptibility *χ*,^47^
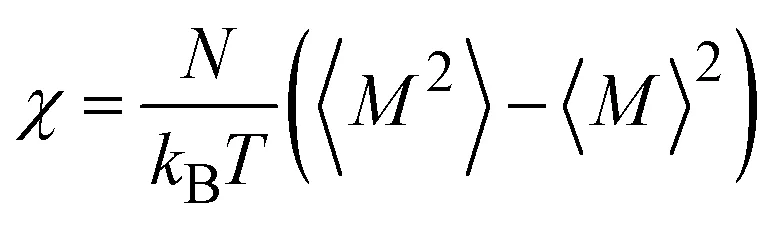
where *N* is the number of spins and *M* is the average magnetization. The use of Numba-accelerated JIT kernels ensured the efficient sampling of the high-dimensional configuration space,^48^ providing a robust estimate of the thermal resilience of the ferromagnetic order in both the pristine and vacancy-engineered phases. The energy mapping uses two spin arrangements: in the ferromagnetic configuration, all Ni and Mn moments are parallel, which is the ground state of both phases.^49^ In the second configuration, the Ni sublattice moments are reversed relative to the Mn sublattice, giving a ferrimagnetic trial state whose net moment does not vanish because the sublattice moments are unequal, with a reduced net moment of about 2 *µ*_B_ compared with 6.00 *µ*_B_ for the ferromagnetic state of the vacancy-engineered phase. The exchange constant follows from 
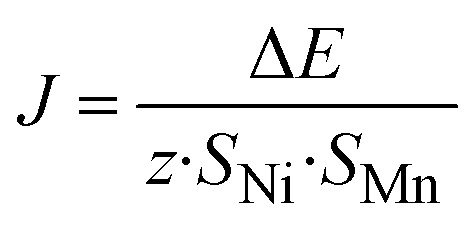
, where Δ*E* is the ferrimagnetic minus ferromagnetic total-energy difference, *z* = 6 is the number of nearest-neighbor Ni–Mn pairs per formula unit in the rock-salt B-site ordering (three Ni–Mn bonds per formula unit, each flipped from parallel to antiparallel at a cost of 2*J S*_Ni_·*S*_Mn_), and *S*_Ni_ = 1 and *S*_Mn_ = 3/2 are the classical spin magnitudes. This yields *J* = 6.5 meV for pristine SNMO and *J* = 19.0 meV for the vacancy-engineered phase, reproducing the Monte Carlo Curie temperatures of 165 K and 480 K, respectively. We then examined this approximation with a Monte Carlo sensitivity analysis. The nearest neighbor coupling was split into vacancy-adjacent and vacancy-distant components (varied by ±20% at a fixed average), and the same sublattice terms, up to 0.05 *J*_eff_, were constrained to a magnitude typical of next-nearest neighbor couplings in ordered double perovskites^50^ and were added to the model. Both modifications shift the predicted Curie temperature of the vacancy-engineered phase by less than 4% (from 480 K to about 465 to 500 K). The reported ordering temperature is therefore a reasonable partitioning of the exchange network, and the single-J treatment serves as an effective model approximation within which the Curie temperatures are model-dependent estimates.

## 3 Results and discussions

### 3.1. Structural analysis

The formation of an oxygen vacancy in Sr_2_NiMnO_6_ (SNMO) is not a localized point-defect event but rather a global structural phase transition driven by the collapse of the coordination polyhedron. As summarized in Table 1 and visualized in Fig. 1, the transition from pristine SNMO to the vacant state (V-SNMO) represents a profound symmetry-lowering reconstruction. SNMO crystallizes in the tetragonal *I*4/*m* space group, where the Ni/Mn octahedra are arranged in a highly ordered, rigid framework (Fig. 1a). However, the removal of apical oxygen atoms abruptly disrupts the octahedral crystal field, eliminating the primary coordination axis and lifting the degeneracy of the in-plane lattice vectors.^51^ This disruption forces the system to undergo a symmetry reduction from *I*4/*m* to the triclinic P1 space group. This transition is driven by elastic strain minimization, as apical oxygen vacancies create an asymmetric electronic environment that induces a local Jahn–Teller-like distortion.^52^ Consequently, the remaining octahedra tilt and rotate to stabilize the under-coordinated metal centers, producing the distorted polyhedral network shown in Fig. 1b.

**Table 1:** DFT-calculated structural, symmetry, and thermodynamic parameters of pristine Sr_2_NiMnO_6_ (SNMO), and its oxygen-deficient counterpart (V-SNMO)

Structural parameters		Sr_2_NiMnO_6_ (tetragonal phase)	Vacant Sr_2_NiMnO_6_
Lattice parameters (Å)	*a*	5.37	5.60
	*b*	5.37	5.53
	*c*	7.84	7.53
*c*/*a*		1.464	1.353
Unit cell volume (Å^3^)		226.24	233.33
Space group		*I*4/*m*	*P*1
Bond lengths (Å)	Sr–O	2.98	2.51
	Ni–O	2.28	2.01
	Mn–O	2.52	1.87
Energy *E*_0_ (eV per atom)		−6.609	−6.658
Formation energy, *E*_f_ (eV per atom)		−1.89	−1.951
Goldschmidt tolerance factor, *T*		0.98	0.995

**Fig. 1:**
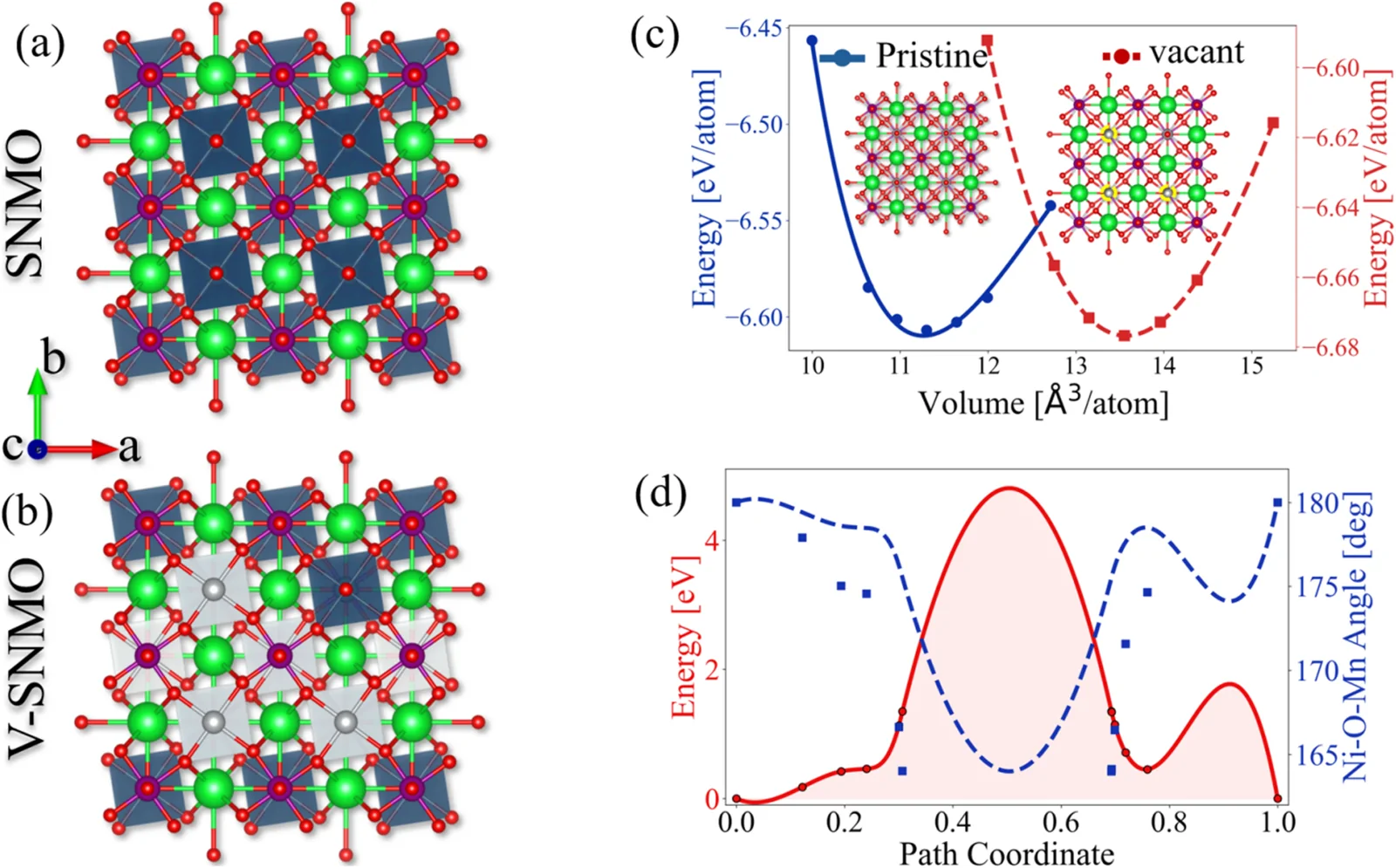
Symmetry breaking and vacancy dynamics in Sr_2_NiMnO_6_. (a) Pristine 2 × 2 × 1 supercell (80 atoms) and (b) oxygen-deficient 2 × 2 × 1 supercell (74 atoms) with the supercell lattice vectors *a*, *b* and *c*, in which the vacancy-adjacent octahedra are shaded gray to identify the six removed oxygen sites and the nearest Ni and Mn neighbors. (c) Volume evolution of pristine *vs.* vacant SNMO and (d) vacancy migration barrier and associated Ni–O–Mn bond-angle distortion.

The macroscopic polyhedral tilting is observed in the anomalous evolution of the lattice parameters. A bifurcation of the basal parameters (*a* = 5.60 Å ≠ *b* = 5.53 Å) and a simultaneous contraction of the *c*-axis (7.84 → 7.53 Å). The increase in *a* and *b* (Table 1) arises from octahedral tilting (Fig. 1b) rather than atomic expansion, as rotation of the octahedra enlarges their projection onto the *ab*-plane.^53^ In contrast, the *c*-axis contracts because removal of the apical oxygen reduces vertical steric repulsion, allowing compression along the *z*-direction. This redistribution of the strain tensor results in a significant reduction of the *c*/*a* ratio from 1.464 to 1.353, confirming that the vacancy transforms the structural anisotropy of the material.^53^

Despite the global lattice expansion, the primary metal–oxygen bonds Sr–O, Ni–O, and Mn–O) contract significantly. This originates from charge compensation following oxygen-vacancy formation, where the released electrons 
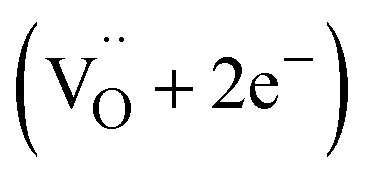
 localize on the Ni/Mn sites, reducing their effective oxidation state and enhancing metal–oxygen bond covalency.^54^ The resulting decrease in electrostatic repulsion allows the oxygen ligands to relax toward the metal centers.^53^ Consequently, the structure exhibits a dual-scale response: local bond contraction driven by electronic redistribution and global lattice expansion induced by octahedral rotation.^54^

Vacancy concentrations at this level are experimentally accessible and reversibly tunable in closely related perovskite oxides. Room-temperature ionic liquid gating converts epitaxial SrCoO_*x*_ films between the perovskite and the brownmillerite SrCoO_2.5_ phase in which 16.7% of the oxygen sites are vacant,^55^ and this topotactic transition has recently been stabilized in freestanding SrCoO_*x*_ membranes.^56^ The trend in lattice expansion observed across this transition (+6.2% in volume for 16.7% vacancies^55^) is consistent with the +3.1% volume change computed here for V-SNMO (Table 1), supporting the experimental realism of the modeled vacancy concentration. The shift in equilibrium volume (∼11.3 → 14 Å^3^ per atom) indicates that the symmetry collapse to *P*1 is the system's primary mechanism for relieving internal chemical pressure. This is further supported by the Goldschmidt tolerance factor *t* increasing toward unity (0.98 → 0.995), suggesting that the system sacrifices point-group symmetry to achieve a more ideal global packing arrangement.^57^

Moreover, the transition to the vacant state is governed by a formidable kinetic bottleneck. The migration physics, mapped *via* the Nudged Elastic Band (NEB) method in Fig. 1d, reveals a dominant energy barrier of ∼4.8 eV. While this high barrier indicates that the vacancies are immobile under purely thermal fluctuations at room temperature, it is a critical requirement for non-volatile memory applications, as it prevents the spontaneous diffusion of vacancies and ensures the long-term stability of the written resistive state.^58^ This penalty is intrinsically coupled to the Ni–O–Mn bond angle *θ*; for an oxygen ion to migrate, the linear superexchange geometry (*θ* ≈ 180°) must collapse to a critical angle of ∼163° to open a transient diffusion channel. The precise synchronization between the energy maximum and this angular minimum demonstrates that the rate-limiting step is the structural reorganization of the superexchange bridge.^59^ Because this angle dictates the orbital overlap between the transition metals and oxygen, the vacancy mobility is fundamentally linked to the magnetic exchange coupling. These findings reveal that the structural degree of freedom controlling ionic conductivity also regulates spin transport, establishing a direct mechanistic basis for the proposed non-volatile switching mechanism.^60^

#### 3.1.1. Decomposition stability

To evaluate the energetic stability of both phases, the formation energy per atom was referenced to standard elemental states, while the vacancy formation energy per defect is defined as 
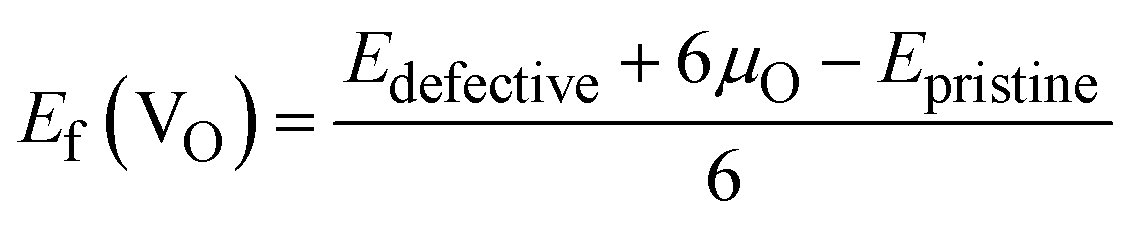
, with *µ*_O_ taken as one half of the total energy of the isolated O_2_ molecule. While a negative formation energy suggests intrinsic stability, it does not preclude the possibility of decomposition into competing phases within the complex Sr–Mn–Ni–O chemical space. To rigorously assess the thermodynamic viability of the host lattice, the decomposition energy (Δ*H*) of pristine Sr_2_MnNiO_6_ (SNMO) against a series of plausible competing oxide phases was evaluated. The decomposition enthalpy is defined by the energy difference between the parent phase and the sum of its decomposition products,Δ*H* = ∑*H*_products_ − ∑*H*_reactants_

A comprehensive survey of potential decomposition pathways was performed, with the most competitive reactions summarized in Table 2. Among these, the lowest-energy pathway of SNMO into SrMnO_3_ and SrNiO_3_ yields a decomposition energy of −0.125 eV per formula unit.

**Table 2:** Calculated decomposition enthalpies for the most competitive phase-separation pathways of pristine Sr_2_MnNiO_6_

Pathways	Δ*H* (per functional unit)
Sr_2_MnNiO_6_ → SrMnO_3_ + SrNiO_3_	−0.125 eV
2Sr_2_NiMnO_6_ → 2 Sr_2_MnO_4_ + 2NiO + O_2_	−0.974 eV
2Sr_2_NiMnO_6_ → 4SrO + 2NiO + 2MnO_2_ + O_2_	−3.974 eV
Sr_2_NiMnO_6_ → 2SrO + Ni + MnO_2_ + O_2_	−4.588 eV
2Sr_2_NiMnO_6_ → 2SrO + 2NiO + 2SrMnO_3_ + O_2_	−2.312 eV
Sr_2_MnNiO_6_ → Sr_2_O_3_ + MnO_2_ + NiO	−5.875 eV

Among the six routes considered, the smallest decomposition enthalpy corresponds to SNMO → SrMnO_3_ + SrNiO_3_ (−0.125 eV per formula unit), the route against which SNMO is most stable, with both products retaining perovskite-related frameworks and requiring only minor structural reorganization, whereas the binary oxide route (−5.875 eV per formula unit) carries the largest driving force for decomposition. While the marginally negative decomposition energy indicates that pristine SNMO is thermodynamically metastable relative to the convex hull of competing oxide phases, its experimental viability is governed by kinetic stability.^61^ The decomposition into compound phases (rather than elemental states) involves significant structural reorganization of the perovskite lattice, which imposes a high kinetic barrier to phase separation.^62^ Furthermore, the strongly negative vacancy formation energy suggests that the system naturally evolves toward the V-SNMO configuration, which represents a stabilized local minimum on the potential energy surface rather than a transient metastable state. Thus, its stability is determined by its energy relative to competing binary and ternary oxides.^63^ The decomposition competitiveness of the parent phase, together with the strongly negative vacancy formation energy, indicates that V-SNMO represents a stabilized local perturbation of the SNMO framework rather than a metastable phase.

#### 3.1.2. Thermodynamic stability

To bridge the gap between the static structural reconstructions and the operational stability of the system, the lattice dynamics of both pristine SNMO and V-SNMO were evaluated based on their phonon dispersion spectra (Fig. 2). The absence of imaginary frequencies across the entire Brillouin zone for both configurations confirms that they correspond to genuine local minima on the potential energy surface.^64^ This dynamic stability confirms that the V-SNMO configuration identified by the EOS and NEB analyses (Fig. 1c and d) is a physically realizable, durable state, rather than a transient intermediate, enabling the structural integrity required for non-volatile memory applications.

**Fig. 2:**
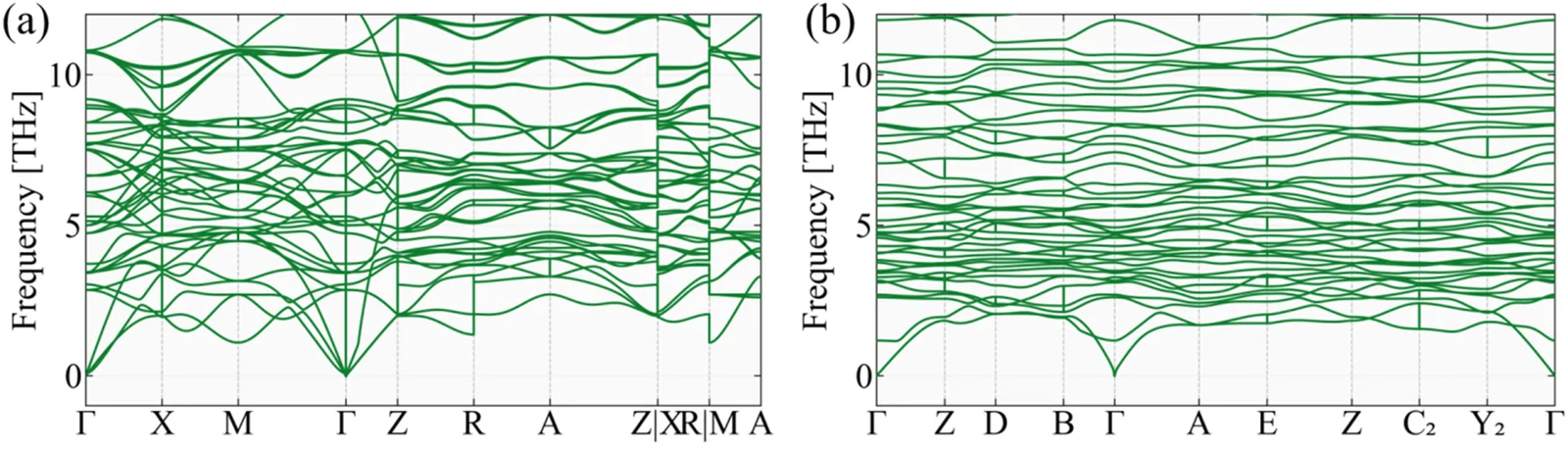
(a) Phonon dispersion of the pristine *I*4/*m* phase. (b) Phonon dispersion of the vacant P1 phase. The transition reveals a pronounced lifting of mode degeneracies and an incipient acoustic softening, providing a dynamic fingerprint of the V_O_-induced symmetry breaking and its associated kinetic facilitators.

The transition from the tetragonal *I*4/*m* to the triclinic *P*1 phase is reflected in the phonon spectra. Pristine SNMO (Fig. 2a) exhibits pronounced mode degeneracies and band crossings at high-symmetry points (*e.g.*, *Z*|*X* and *R*|*M*), imposed by crystal symmetry. In contrast, V-SNMO (Fig. 2b) displays a denser, smoother phonon manifold with closely spaced branches. This lifting of mode degeneracies arises from vacancy-induced local distortion and the collapse of the Ni–O–Mn bond angle, producing peak splitting and enhanced spectral density, particularly in the 3–8 THz range, as a vibrational fingerprint of the symmetry reduction to the *P*1 phase.^65^

Beyond symmetry-induced mode splitting, the lattice dynamics reveal a direct coupling between vibrational softening and oxygen-ion migration. V-SNMO (Fig. 2b) exhibits a pronounced softening of the acoustic branch, approaching near-zero frequency at the intermediate *Γ*-point (between B and A), indicating enhanced lattice flexibility associated with octahedral tilting.^63^ This soft mode is the dynamical counterpart of the migration pathway identified by the NEB analysis (Fig. 1d), where lattice softening along the tilting coordinate reduces the structural reorganization energy required for oxygen-ion hopping. The combination of dynamical stability, symmetry-induced degeneracy lifting, and acoustic softening indicates that the vacancy-engineered phase supports enhanced ion mobility while remaining structurally stable.

### 3.2. Electronics analysis

To uncover the microscopic basis for the spintronic performance of Sr_2_NiMnO_6_ (SNMO), the spin-polarized electronic structures of the pristine and oxygen-vacancy-engineered (V-SNMO) phases were investigated (Fig. 3). The pristine structure (Fig. 3a) exhibits a striking spin-channel asymmetry. The spin-up channel is characterized by a wide, well-defined energy gap of ∼1.5 eV between the valence band maximum (VBM) and the conduction band minimum (CBM), indicating a robust semiconducting state. In contrast, the spin-down channel is dominated by a weakly dispersive, nearly flat band (highlighted in orange in the C–Z bands) that resides in close proximity to the Fermi level (*E*_F_). No pristine band crosses the Fermi level in either spin channel; the spin-up gap is given above, while the spin-down gap amounts to 0.2 eV. This narrow-gap behavior is attributed to the localized nature of the Ni/Mn e_g_ orbital manifold, which suppresses kinetic hopping and creates a quasi-degenerate state at *E*_F_.^15^ This asymmetry is the governing factor for efficient spin-selective transport, a fundamental requirement for enhancing spin-injection efficiency in magnetic tunnel junctions (MTJs).^3^ The three-dimensional Fermi surface reconstruction (Fig. 3b), generated from bands 281–283 in the first Brillouin zone, further substantiates the electronic structure. The Fermi surface consists of small, isolated pockets localized near the Brillouin zone boundary, reflecting the low carrier density and the narrow-gap character of the minority-spin channel. In contrast, the complete absence of Fermi surface sheets in the majority-spin channel confirms full spin polarization.^3,59^ Furthermore, the proximity of the spin-down band to *E*_F_ renders the electronic state highly susceptible to structural perturbations, providing a strategic pathway for tuning the material's conductivity through oxygen-vacancy engineering.^60^

**Fig. 3:**
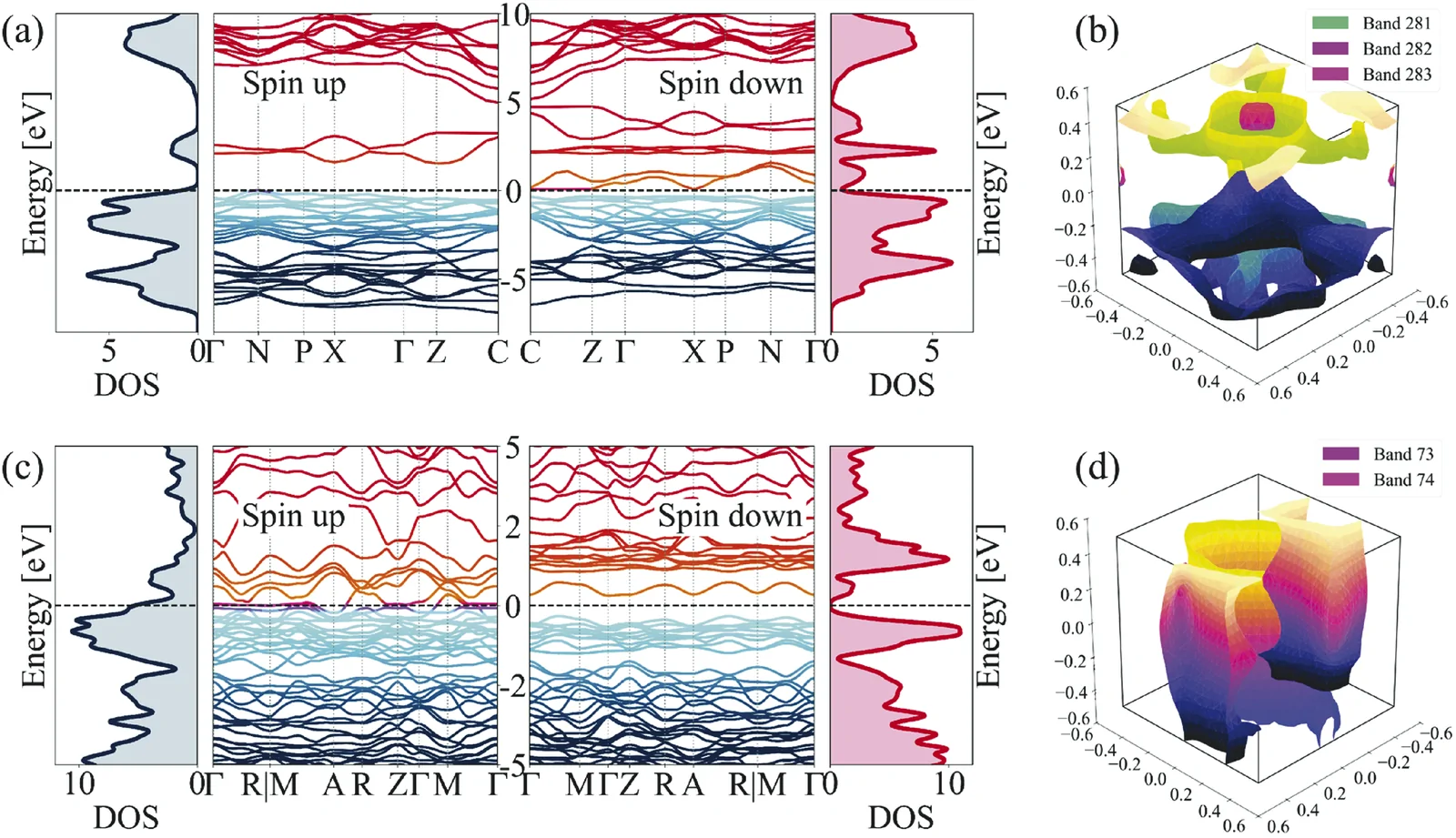
Spin-polarized electronic structure and Fermi surface topology of pristine and V-SNMO. (a and c) Band structures and DOS showing the transition from a spin-asymmetric semiconductor (pristine) to a half-metal (vacancy-engineered). (b and d) Corresponding 3D representations of the electronic states at *E*_F_, illustrating the reconfiguration of the Fermi surface from localized pockets to enlarged, warped and interconnected sheets upon the introduction of oxygen vacancies. All calculations correspond to the ferromagnetic ground state on the fully relaxed 2 × 2 × 1 supercells (zone-folded representation).

The introduction of an oxygen vacancy (V_O_) markedly perturbs the electronic landscape of SNMO, inducing a spin-asymmetric reconfiguration of the band structure (Fig. 3c). Following the Kröger–Vink formalism 
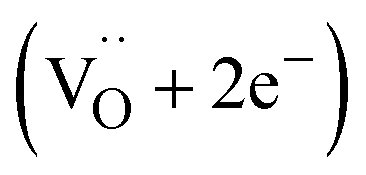
, the vacancy acts as a double-donor, injecting two electrons into the system.^66,67^ These carriers preferentially populate the spin-up defect states (highlighted in magenta), triggering a collapse of the spin-up gap and driving a selective semiconductor-to-metal transition (SMT) in the majority channel.^68^

This divergent behavior is attributed to the localization of the donor electrons into vacancy-induced Ni^4+^/Ni^2+^-like electronic character, as shown in Fig. 4, which strengthens the local magnetic moments and enhances the double-exchange-mediated exchange splitting.^19^ The resulting increase in the exchange energy pushes the spin-down conduction band further above the Fermi level (*E*_F_), effectively insulating the minority channel while the majority channel becomes itinerant.^19^

**Fig. 4:**
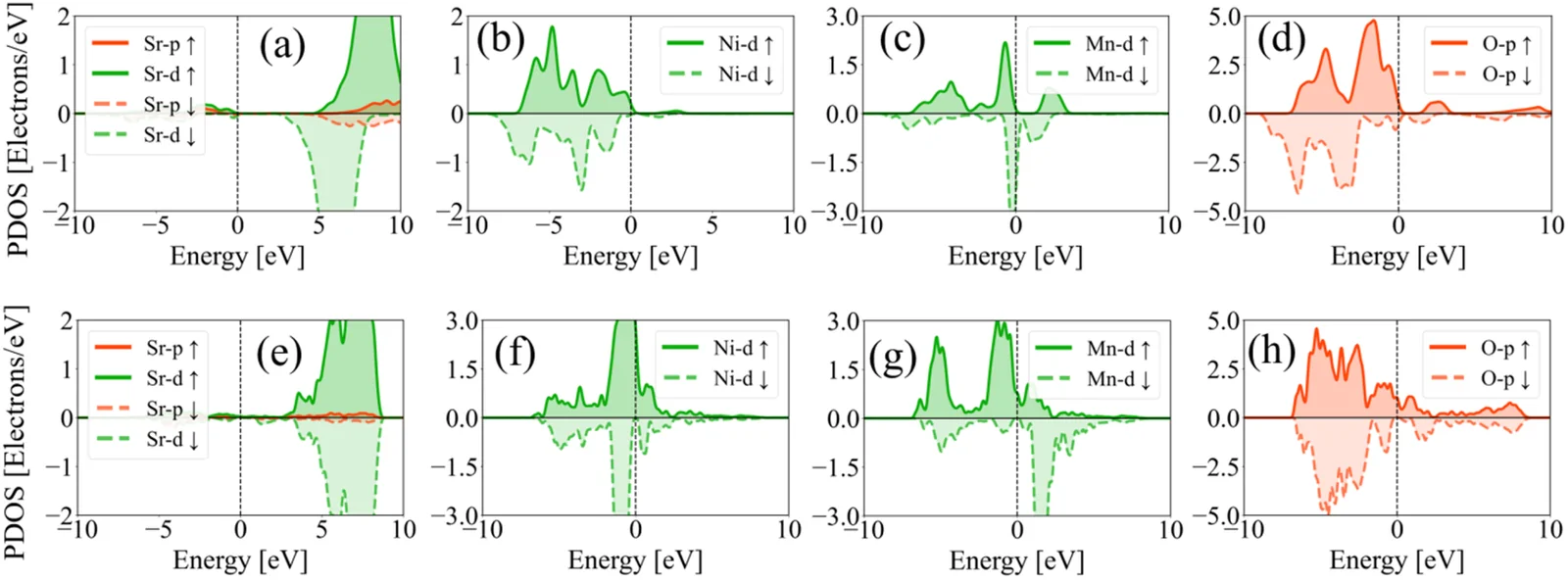
Spin-polarized PDOS of the pristine and vacancy-engineered Sr_2_NiMnO_6_. (a–d) Pristine phase exhibiting a spin-asymmetric semiconducting gap dominated by the Mn e_g_ states. (e–h) V-SNMO phase showing the collapse of the spin-up gap due to Ni-site charge localization. The resulting spectral asymmetry confirms the transition from a near-half-metallic to a true half-metallic electronic configuration.

This transition is manifested in the 3D Fermi surface topology (Fig. 3d), where the emergence of enlarged, warped, interconnected sheets (Bands 73–74) is restricted exclusively to the spin-up channel. This topology confirms the high density of itinerant carriers in a single spin species, a hallmark of half-metallicity. From a functional perspective, this vacancy-driven SMT provides the fundamental physical basis for resistive-state bistability.^58^ This mechanism suggests that oxygen vacancy (V_O_) migration under an external bias can reversibly switch the material between distinct electronic and magnetic phases, making V-SNMO a promising active layer for oxygen-vacancy-mediated non-volatile memory devices.^58^

To resolve the microscopic origin of the observed electronic transitions, the atom- and orbital-projected density of states (PDOS) for both the pristine and vacancy-engineered systems were analyzed (Fig. 4). In the pristine phase, the Sr p/d states (Fig. 4a) are located far from the Fermi level (*E*_F_ > 3 eV), confirming that Sr acts as an electrostatic stabilizer in a purely ionic Sr^2+^ configuration, contributing negligibly to the transport properties.^3^

The electronic manifold near *E*_F_ is governed by the hybridization of the transition-metal 3d and O 2p orbitals. In pristine SNMO, the Ni 3d states (Fig. 4b) exhibit a robust exchange splitting, with the majority of the spectral weight pushed away from *E*_F_, consistent with a stable low-spin Ni^4+^ (t^6^_2g_/d^6^) configuration.^50^ Conversely, the Mn 3d PDOS (Fig. 4c) reveals a sharp, narrow-band feature in the spin-down channel pinned near *E*_F_. This narrow Mn e_g_ resonance is the fundamental origin of the small spin-down gap of 0.2 eV identified in the band structure (Fig. 4a), resulting from the crystal-field splitting under octahedral coordination.^50^ The strong overlap between O 2p (Fig. 4d) and the Ni/Mn 3d states in the −7 to −2 eV range confirms the existence of a strong p–d hybridization, which mediates the Ni–O–Mn superexchange interaction stabilizing the ferromagnetic order.^50,69^

The introduction of oxygen vacancies induces a qualitative reconfiguration of this orbital landscape (Fig. 4e–h). The most striking feature is the emergence of an intense, sharp in-gap resonance in the Ni 3d channel (Fig. 4f) that straddles *E*_F_. This is the direct spectral fingerprint of the V_O_-induced donor electrons preferentially localizing on the Ni sites, signaling a predominantly Ni-centered reduction from formal Ni^4+^ toward Ni^2+^-like character.^70^ Simultaneously, the Mn 3d states (Fig. 4g) exhibit an enhanced spin-up spectral weight shifted away from *E*_F_, reflecting a reinforcement of the exchange splitting *via* the double-exchange mechanism. The corresponding depletion of O 2p intensity near *E*_F_ (Fig. 4h) confirms the local loss of hybridization at the vacancy site. This orbital analysis provides a rigorous basis for the vacancy-driven SMT: the metallization is primarily Ni-centered, while the Mn sites maintain and enhance their localized magnetic character.^50^

To observe hybridization in Sr_2_NiMnO_6_ (SNMO), the interplay between the transition-metal 3d and O 2p manifolds was analyzed (Fig. 5). The magnetic ground state is governed by the asymmetric orbital occupancy of the Ni^4+^(d^6^,t^6^_2g_e^0^_g_) and Mn^4+^(d^3^,t^3^_2g_e^0^_g_) ions. This configuration creates a unique orbital-selective environment where the exchange interaction is partitioned into two simultaneous, cooperative channels (Fig. 5b).^50^

**Fig. 5:**
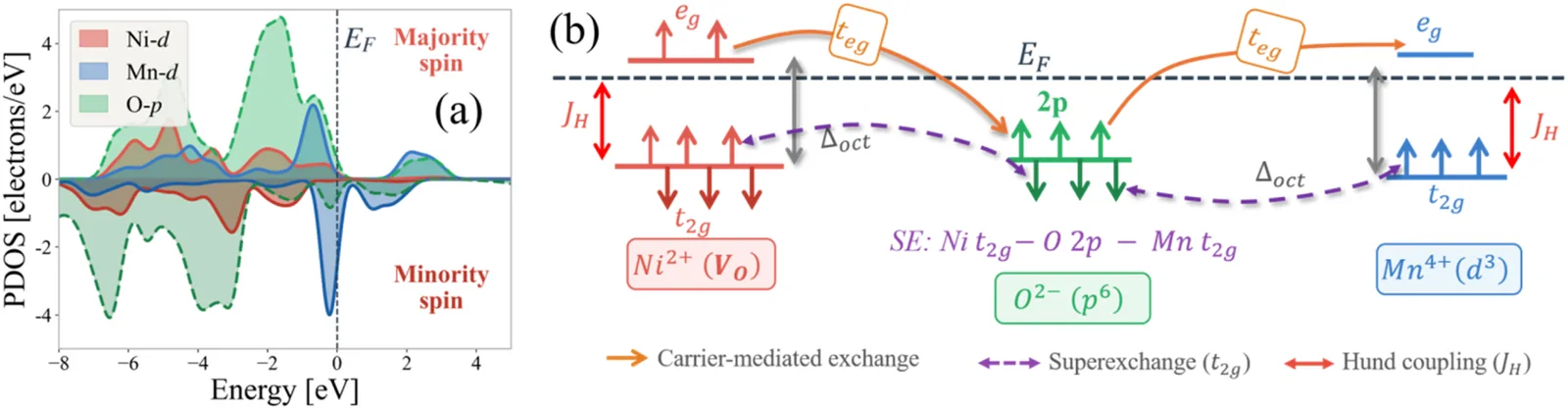
Electronic structure and magnetic exchange model of Sr_2_NiMnO_6_. (a) Total and orbital-projected DOS revealing the spin-polarized electronic manifold. (b) Schematic of the dual-channel exchange model, illustrating the role of Hund's coupling and crystal-field splitting in driving the Ni–O–Mn ferromagnetic coupling through superexchange, with a possible carrier-mediated channel enabled by vacancy-induced Ni reduction.

The first channel is the t_2g_-O 2p-t_2g_ superexchange (SE) interaction. With a filled Ni t_2g_ shell and a half-filled Mn t_2g_ shell, virtual hopping is Pauli-forbidden. According to the Goodenough–Kanamori–Anderson (GKA) rules, this 180° M–O–M configuration favors ferromagnetic (FM) coupling between the t_2g_ cores.^71^ This localized SE interaction forms the magnetic backbone of the system, consistent with the sharp, high-intensity Mn d minority-spin peak near *E*_F_ shown in Fig. 5a.

Simultaneously, the system supports an e_g_-O 2p-e_g_ double-exchange (DE) pathway. The unpaired Ni e_g_ electrons can virtually hop into the empty Mn e_g_ states, maximizing kinetic energy when the Ni and Mn spins are aligned parallel, as enforced by strong intra-atomic Hund's coupling (*J*_H_).^72^ Simultaneously, the system supports a double exchange (DE) pathway when the vacancy-induced Ni-centered reduction supplies e_g_ electrons, which can virtually hop into the empty Mn states, maximizing kinetic energy when the Ni and Mn spins are aligned parallel, as enforced by strong intra-atomic Hund coupling (*J*_H_). Such a carrier-mediated channel, together with the surviving superexchange paths, is consistent with the half-metallic character of the vacancy-engineered phase^72^ and the dispersive bands observed in the band structure (Fig. 3a). The strengths of both the SE and DE interactions are governed by the octahedral crystal-field splitting (Δ_oct_) and the resulting p–d hybridization.^50^ The pronounced overlap between the O 2p and Ni/Mn 3d states over the −6 to 0 eV range (Fig. 5a) indicates strong covalent mixing, enabling the O 2p orbitals to mediate both localized and itinerant exchange interactions.^50^

This dual-exchange framework explains the persistence of ferromagnetism under oxygen-vacancy engineering.^73^ While V_O_ modifies the e_g_ double-exchange channel by altering the carrier density and enhancing carrier localization, consistent with the increased effective mass in Table 3, the t_2g_ superexchange network remains largely preserved.

**Table 3:** Spin-resolved effective masses and Bader charge analysis accompanying the vacancy-induced semiconductor-to-metal transition in Sr_2_NiMnO_6_

State	Effective mass		Element	Bader electrons (e^−^)	Bader charge (*e*)	Atomic volume (Å^3^)
	Spin up	Spin down				
Pristine	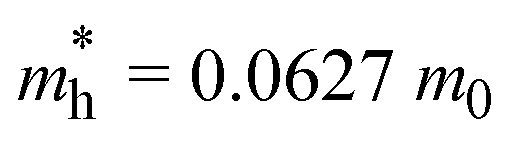	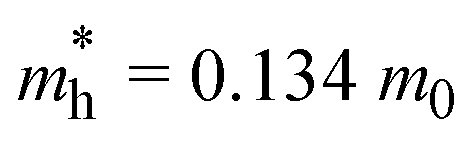	Sr	8.4164	+1.5836	15.0742
			Ni	8.4816	+1.5184	6.9385
	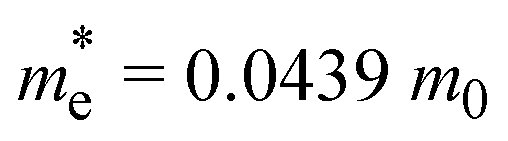	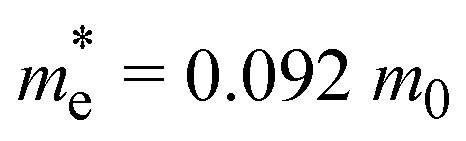	Mn	11.1406	+1.8594	6.8510
			O	7.0908	−1.0908	11.5310
O vacant	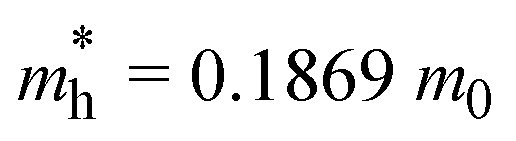	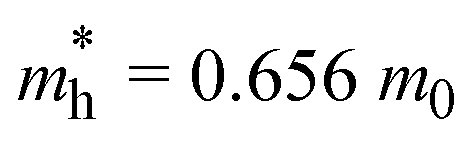	Sr	8.4484	+1.5516	16.6172
			Ni	9.0680	+0.9320	12.7528
	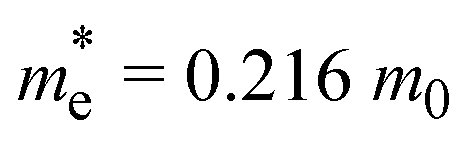	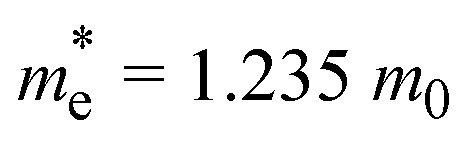	Mn	11.4556	+1.5444	10.2988
			O	7.2399	−1.2399	13.0882

The spin-resolved carrier effective masses (m*) were extracted using a second-order parabolic fit to the band curvature at the *Γ* point, and Bader analysis (Table 3) provides a quantitative foundation for the electronic transitions in the band structure. In pristine SNMO, the significant asymmetry in M*, where spin-up carriers are substantially lighter (*m*_h_ ≈ 0.06 *m*_0_, *m*_e_ ≈ 0.04 *m*_0_) than spin-down carriers, is a direct consequence of the band curvature analyzed in Fig. 3a. The high dispersion of the spin-up manifold yields low effective masses, whereas the narrow, Mn-derived flat band pinned at *E*_F_ results in heavier carriers, consistent with the localized, strongly correlated nature of the spin-down channel.^72,73^ This band is characterized by a strong hybridization between the Ni 3 d-e_g_ and O 2p orbitals, which results in a highly dispersive electronic state and, consequently, a low effective mass.

The introduction of oxygen vacancies triggers a pronounced increase in m* across both spin channels, with the spin-down electron mass reaching 1.235 *m*_0_. This increase indicates a transition toward band flattening and enhanced carrier localization, despite the emergent metallicity.^73^ This phenomenon suggests that the vacancy-induced metallization is not a simple rigid-band shift but is mediated by the formation of narrow, localized Ni-centered defect states (Fig. 4f). The accompanying increase in m* is a hallmark of defect-mediated transport, where carriers exhibit polaronic character due to strong coupling with the distorted local lattice.^70^

In the pristine phase, the formal valences of Ni (+1.52 *e*) and Mn (+1.86 *e*) (Table 3) reflect stable Ni^4+^/Mn^4+^-like configurations. Upon vacancy formation, the Ni Bader charge decreases sharply to +0.93 *e*, while the Mn charge remains relatively stable (+1.54 *e*). This indicates that the two electrons released by the 
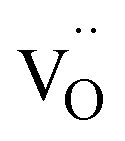
 center are preferentially captured by the Ni sites, driving a Ni^4+^to Ni^2+^-like reduction.^74^ The physical manifestation of this charge transfer is evidenced by the dramatic expansion of the Ni atomic volume (6.94 → 12.75 Å^3^), which corresponds to the increased ionic radius of the reduced Ni species.

### 3.3. Magnetic analysis

The fundamental viability of any spintronic material depends on the robustness of its magnetic ground state against competing ferrimagnetic (FiM) alignments. To quantify this stability, the total energy as a function of volume is analyzed for both ferromagnetic (FM) and ferrimagnetic (FiM) configurations. As shown in Fig. 6a and b, the energy landscape for both pristine and V-SNMO reveals that the FM state (blue curve) is the absolute ground state, as it maintains a lower total energy compared to the FiM state (red dashed curve) across the entire volume range. The energy difference represents the stability of the ferromagnetic order. The fact that this gap remains pronounced in the vacant state (Fig. 6b) indicates that the introduction of oxygen deficiencies does not destabilize the ferromagnetic order but rather shifts the equilibrium volume of the stable phase.

**Fig. 6:**
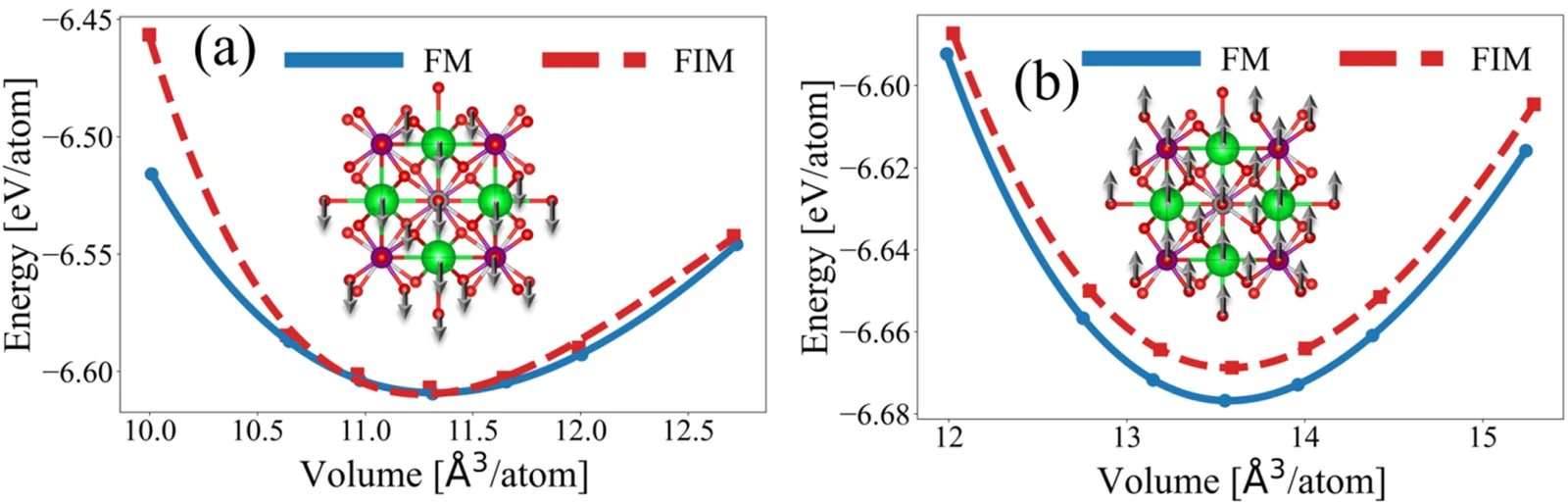
Magnetic ground state stability and structural evolution of Sr_2_NiMnO_6_. (a) Total energy as a function of volume for the ferromagnetic (FM) and ferrimagnetic (FiM) configurations of pristine SNMO, with the corresponding structural model highlighting the ordered polyhedral framework. (b) Total energy *vs.* volume for the oxygen-deficient V-SNMO phase, demonstrating the shift in equilibrium volume and the persistence of the FM ground state.

The most striking transformation occurs in the magnetic manifold upon vacancy formation. According to the data presented in Table 4, the total magnetic moment undergoes a substantial jump from −2.97 *µ*_B_ in the pristine phase to a fully saturated 6.00 *µ*_B_ in the V-SNMO phase. This is not merely a quantitative increase but a qualitative shift in the electronic configuration. In pristine SNMO, the magnetic moment is primarily driven by the Mn site (−2.48 *µ*_B_), while the Ni site contributes minimally (−0.34 *µ*_B_), suggesting an unsaturated magnetic ground state. In contrast, V-SNMO exhibits a total magnetic moment of 6.00 *µ*_B_, consistent with the high-spin configuration of Ni^2+^ (d^8^, *S* = 1) and Mn^4+^ (d^3^, *S* = 3/2). The combined spin quantum number (*S*_Ni_ + *S*_Mn_ = 5/2) therefore yields a spin-only moment of 5 *µ*_B_ per formula unit, with the difference fro5m the 6.00 *µ*_B_ DFT value carried by the ligand O 2p polarization, giving 6 *µ*_B_, confirming that oxygen-vacancy formation induces a valence-driven reduction that maximizes the spin polarization of the transition-metal 3d manifold.^50^

**Table 4:** Electronic structures, charge analysis, and spin-resolved magnetic moments of pristine and oxygen-deficient Sr_2_NiMnO_6_

Compound	Charge spilling	Species	*s*	*p*	*d*	*f*	Total	Mulliken charge	Formal ionic charge	Effective valence	Hirshfeld charge	Spin magnetic moment, *µ*_S_	Total magnetic moment, *µ*_B_
SNMO	0.19%	Sr	2.11	6.30	0.67	0.00	9.08	0.92	2	1.08	0.25	−0.02	−2.97
		Ni	0.42	0.70	8.27	0.00	9.39	0.61	4	1.59	0.31	−0.34	
		Mn	2.00	6.00	5.16	0.00	13.16	1.84	4	2.16	0.38	−2.48	
		O	1.87	4.84	0.00	0.00	6.71	−0.71	−2	1.29	−0.22	−0.02	
V-SNMO	0.21%	Sr	2.04	5.89	0.22	0.00	8.15	1.85	2	0.15	0.50	0.03	6.00
		Ni	0.39	0.46	8.38	0.00	9.23	0.77	2	1.23	0.39	0.29	
		Mn	0.34	6.49	5.13	0.00	11.96	3.04	4	0.96	0.64	3.03	
		O	1.30	2.85	0.00	0.00	4.15	−1.85	−2	0.15	−0.57	0.01	

In the pristine lattice, the FM order is mediated by Ni (e_g_)–O (2p)–Mn (t_2g_) superexchange (SE) interactions.^9,71^ While the SE provides the basic FM backbone, it is relatively weak, as evidenced by the lower total moment. The removal of the oxygen bridge in V-SNMO disrupts the SE pathway, but this is more than compensated for by the injection of two donor electrons into the system. These electrons populate the majority-spin channel, consistent with an enhancement of carrier-mediated exchange resembling a Zener-type double-exchange picture, though the itinerant-hopping mechanism itself is not directly demonstrated by the present calculations.^10^ This carrier-mediated exchange picture is favored over the weaker SE pathway in driving ferromagnetism because it would maximize the kinetic energy of the itinerant electrons when the Ni and Mn core spins are aligned parallel. Consequently, the V-SNMO phase is not only more highly magnetized but also more “robustly” ferromagnetic.

To evaluate the operational viability of SNMO for room-temperature spintronic applications, Monte Carlo simulations were performed to determine the Curie temperature (*T*_C_) and the associated thermodynamic observables, as shown in Fig. 7. The temperature-dependent normalized magnetization, *M*/*M*_sat_ (Fig. 7a and e), reveals a marked enhancement in thermal stability upon oxygen-vacancy engineering. Monte Carlo simulations predict a Curie temperature of *T*_C_ ≈ 165 K for pristine SNMO. In contrast, the vacancy-engineered phase exhibits a significantly enhanced *T*_C_ ≈ 480 K (Fig. 7e), reflecting an enhancement of exchange coupling consistent with a shift from a superexchange (SE)-dominated toward a more carrier-mediated magnetic regime, though the itinerant double-exchange (DE) mechanism itself is inferred rather than directly demonstrated. Because *T*_C_ scales with the exchange integral *J*, this increase indicates that vacancy-induced itinerant electrons strengthen the exchange coupling between the Ni and Mn sublattices, stabilizing ferromagnetic order well above room temperature.^21^

**Fig. 7:**
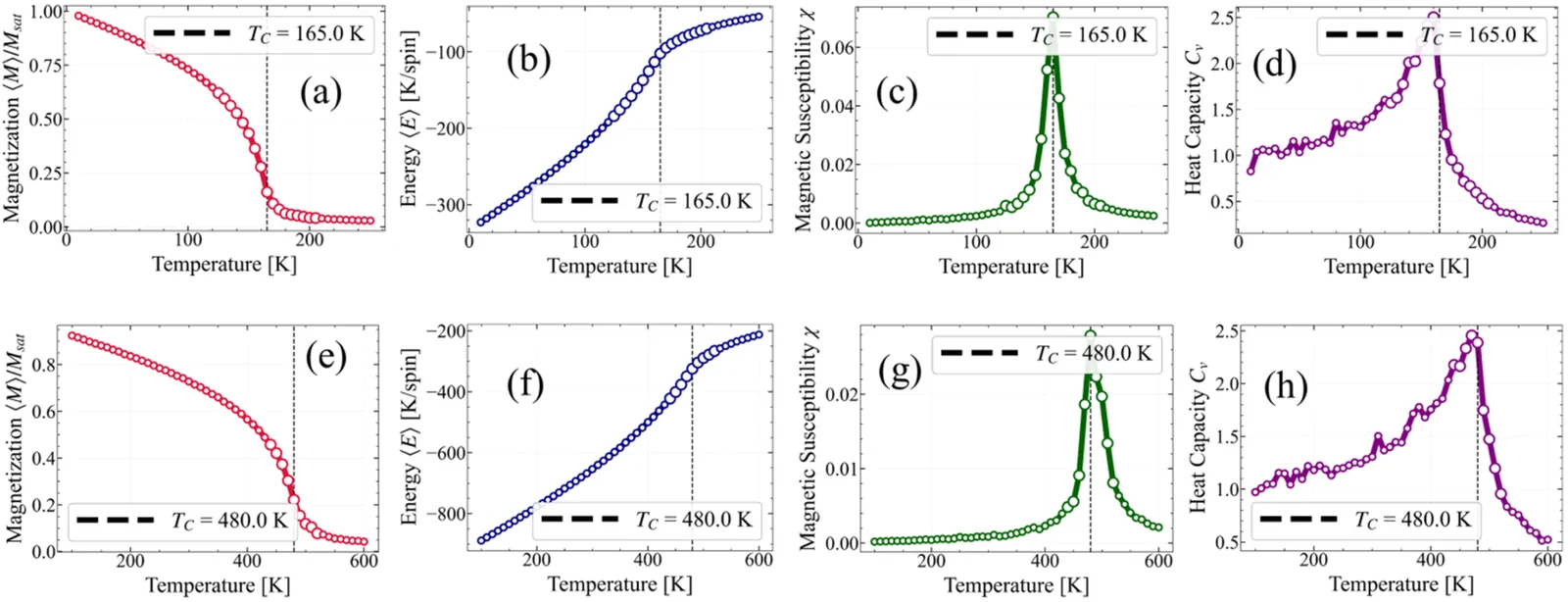
Temperature dependence of the magnetic observables for (top row) pristine SNMO and (bottom row) V-SNMO: (a and e) normalized magnetization *M*/*M*_sat_ showing the transition from a cryogenic magnet (*T*_C_ = 165.0 K) to a room-temperature ferromagnet (*T*_C_ = 480.0 K); (b and f) internal energy *E* per spin, illustrating the increase in exchange stiffness in the vacant phase; (c and g) magnetic susceptibility *χ*, exhibiting singular peaks at the Curie temperature; and (d and h) heat capacity *C*_v_ showing the characteristic λ-anomaly associated with the second-order phase transition.

The enhanced magnetic stability is further supported by the internal energy *E* profiles (Fig. 7b and f). V-SNMO exhibits a substantially lower internal energy (≈−900 K per spin) than pristine SNMO (≈ −320 K per spin), indicating stronger ferromagnetic exchange. Within the Heisenberg model,^23^*E* ∝ −*J*∑**S**_*i*_·**S**_*j*_. Thus, the deeper energy minimum reflects an increased exchange interaction, elevating *T*_C_. The magnetic susceptibility (*χ*) (Fig. 7c and g) and heat capacity (*C*_v_) (Fig. 7d and h) both exhibit sharp peaks at *T*_C_, characteristic of a second-order ferromagnetic–paramagnetic transition. In V-SNMO, the susceptibility peak at 480 K marks the onset of critical spin fluctuations, while the λ-type anomaly in *C*_v_ reflects the associated magnetic entropy change. The well-defined *C*_v_ peak further confirms that the vacancy-induced ferromagnetism is a collective long-range state rather than a localized defect-driven effect.

From a device perspective, these thermodynamic results have important implications for non-volatile spintronic memory. For STT-MRAM, the thermal stability factor 
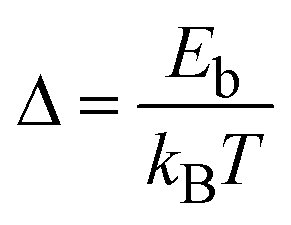
,^2^ must be sufficiently large to suppress thermally activated magnetization reversal. Because pristine SNMO is paramagnetic at room temperature, it cannot support stable magnetic data storage. In contrast, V-SNMO remains ferromagnetic at 300 K, ensuring thermally stable magnetic states. Moreover, the ∼300 K increase in *T*_C_ achieved through oxygen-vacancy engineering suggests that magnetic properties can be electrically tuned by controlling the vacancy concentration, providing a route toward integrated non-volatile resistive switching with spin-based functionality.

### 3.4. Thermoelectric analysis

To evaluate the potential of SNMO for spin-caloritronic applications, the spin-resolved Seebeck coefficient (*S*), electrical conductivity (*σ*/*τ*), electronic thermal conductivity (*κ*_e_/*τ*), power factor (PF/*τ*), and electronic figure of merit (*ZT*_e_) using semi-classical Boltzmann transport theory were computed (Fig. 8).^45^ The Seebeck coefficient (Fig. 8a) reveals a striking divergence in the temperature dependence between the two spin channels. In pristine SNMO, *S*_↑_ increases monotonically with temperature, while *S*_↓_ exhibits a rapid decay. This opposite-sign temperature dependence is a direct manifestation of the Mott relation 
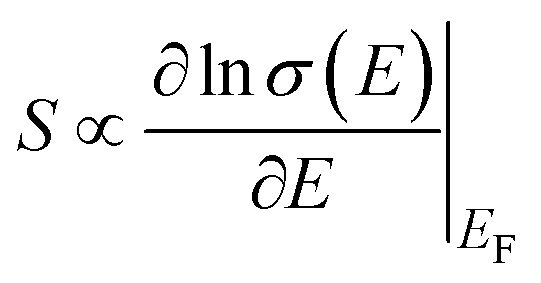
, reflecting the asymmetry in the density of states (DOS) slope at the Fermi level.^72^ Specifically, the narrow, flat Mn d-derived band pinned near *E*_F_ (Fig. 8c) produces a large but rapidly saturating DOS slope, leading to a high *S*_↓_ at low temperatures that vanishes as thermal broadening samples the flatter regions of the band. Conversely, the light, dispersive spin-up states sustain a slope that increases with *T*. Upon vacancy formation, the collapse of *S*_↓_ further confirms the widening of the spin-down gap, which suppresses the energy derivative of the conductivity in the minority channel.^72^

**Fig. 8:**
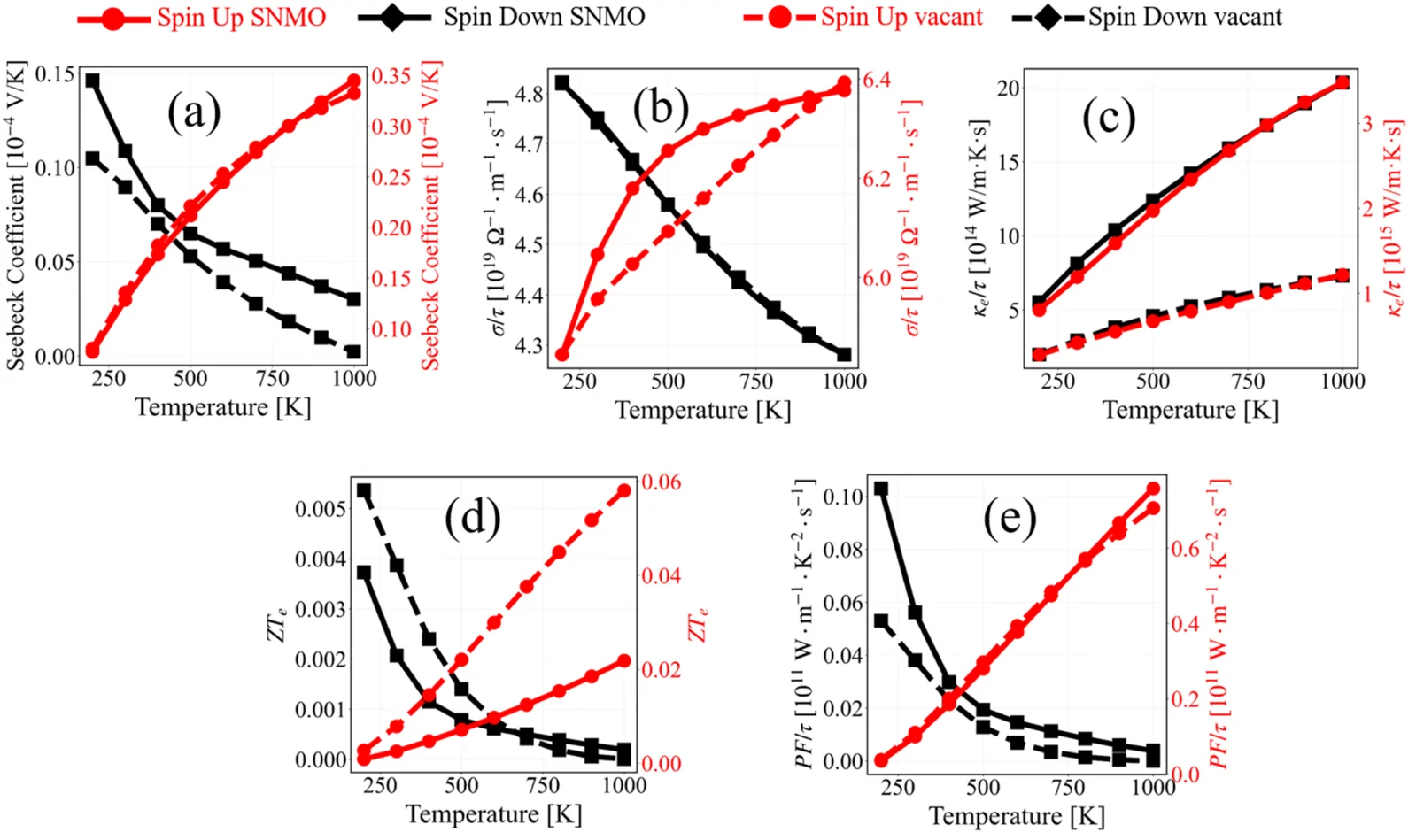
Spin-polarized thermoelectric transport properties of pristine and V-SNMO. Evolution of (a) Seebeck coefficient, (b) electrical conductivity, (c) electronic thermal conductivity, (d) electronic figure of merit, and (e) power factor across the 200–1000 K range. The results illustrate the modification of the spin-dependent transport manifold upon vacancy introduction, characterized by a selective suppression of the minority-spin channel and the enhancement of the majority-spin caloritronic response.

The electrical conductivity (Fig. 8b) further underscores this spin-selective behavior. The spin-up channel exhibits metallic-like behavior (*σ*/*τ* ∝ *T*), while the spin-down channel shows a semiconducting-like decrease. Notably, the V-SNMO system exhibits a reduction in *σ*_↑_/*τ* relative to the pristine phase at intermediate temperatures. This is a transport-level confirmation of the increased effective masses calculated in Table 3 (*M*_h_: 0.06 → 0.19 *m*_0_); the V_O_-induced metallization is not a simple rigid-band shift. The dramatic increase in the carrier effective mass (*M*_h_: 0.06 → 0.19 *m*_0_) calculated in Table 3, combined with the emergence of narrow Ni-centered resonances in the PDOS, is consistent with possible polaronic character.^70^ These states increase carrier inertia and provide a microscopic origin for the enhanced scattering observed in the transport manifold. The electronic thermal conductivity (Fig. 8c) closely follows the Wiedemann–Franz relation, *κ*_e_ = *L*_0_*σT*.^72^ In the pristine phase, *κ*_e,↑_ and *κ*_e,↓_ are markedly different, implying that heat is carried preferentially by one spin species. Upon vacancy formation, these two channels nearly converge. This indicates that vacancy engineering “equilibrates” thermal transport across spin channels, effectively reducing the imbalance in the spin-heat current. This result is critical for thermal management in nanoscale MRAM cells, as it suggests that vacancies can be used to tune local heat dissipation without destroying the spin polarization of the electrical current.

The culmination of these effects is observed in the power factor and *ZT*_e_ (Fig. 8d and e). At high temperatures, a complete bifurcation of the transport channels occurs: the vacancy-engineered state sustains a high *ZT*_e,↑_, while *ZT*_e,↓_ collapses. It should be emphasized that the figure of merit reported here corresponds strictly to the electronic contribution, *ZT*_e_ = *S*^2^*σT*/*κ*_e_, evaluated within the semi-classical Boltzmann transport framework.^72^ The full thermoelectric figure of merit, *ZT* = *S*^2^*σT*/(*κ*_e_ + *κ*_L_), additionally requires the lattice thermal conductivity (*κ*_L_), which is not captured in BoltzTraP2 calculations and will reduce the actual *ZT* in practical devices; therefore, the present values serve as computational upper bounds. Oxygen-vacancy engineering does not merely increase carrier density; it selectively modulates the transport manifold, suppressing the minority spin channel while optimizing the majority channel. The crossover near *T* ≈ 400–500 K marks the thermal threshold where this spin-selective bifurcation becomes the dominant transport mechanism. The Power Factor (PF = *S*^2^*σ*) (Fig. 8e) represents the material's capacity to convert a temperature gradient into electrical power (or *vice versa*). Enhancement of PF_↑_ in the vacant system was observed, while PF_↓_ collapses. The vacancy does not merely add carriers; it optimizes the *S*^2^*σ* product selectively for the spin-up channel. This predicted ‘spin-selective power enhancement,’ rooted in the reconfiguration of the spin-polarized DOS, suggests a potential driving force for thermally assisted spin-torque switching, where a high PF in a single spin channel allows for the generation of a strongly spin-polarized current *via* a modest thermal gradient.^2^ This transition from a quasi-half-semiconductor to a half-metallic transport regime provides a physical basis for thermally addressable memory. Reversible switching between spin-symmetric (pristine) and highly spin-polarized (V-SNMO) transport *via* oxygen-vacancy migration enables a dual-mode read/write mechanism, providing an additional control axis through thermopower modulation for thermally assisted spin-torque switching with a potentially large spin-dependent transport contrast.^58^

## 4 Conclusion

In this study, it has been demonstrated that oxygen-vacancy engineering in Sr_2_NiMnO_6_ is not merely a defect-induced perturbation, but a fundamental driver of a coupled structural, electronic, and magnetic phase transition. The removal of oxygen atoms triggers a symmetry collapse from the tetragonal *I*4/*m* to the triclinic P1 phase, which serves as the structural prerequisite for reconfiguration of the transition-metal 3d manifold. This transition is governed by the V_O_-induced injection of electrons into the Ni sites, driving a Ni^4+^ toward a Ni^2+^ redox process. This creates a spin-selective semiconductor-to-metal transition (SMT) that transforms the system from a spin-asymmetric semiconductor into a half-metal. The resulting high-spin configuration maximizes the spin polarization of the conduction channel, as is further validated by the emergence of polaronic Ni-centered resonances at the Fermi level. The dramatic surge in the total magnetic moment from −2.97 *µ*_B_ to a saturated 6.00 *µ*_B_, alongside the elevation of the Curie temperature from 165 K to 480 K, marks a fundamental shift from a weak superexchange-dominated regime toward enhanced carrier-mediated exchange consistent with, but not directly proven to be, a Zener double-exchange mechanism. Crucially, the observed increase in carrier effective mass (reaching 1.235 *m*_0_) is consistent with possible polaronic character. Such localization, coupled with the spin-selective bifurcation of the thermoelectric power factor, indicates that the metallization is not a simple rigid-band shift but a reconfiguration of the electronic landscape.

The high *T*_C_ (480 K) ensures that the ferromagnetic order remains thermally stable well above room temperature, while the complete spin polarization at the Fermi level of the half-metallic state optimizes spin-injection efficiency for magnetic tunnel junctions. Furthermore, V_O_ migration and the transition from a semiconducting to a half-metallic state suggest a dual-mode read/write mechanism *via* the massive contrast in spin-polarized transport. By integrating resistive switching with high-temperature spin-polarized itinerancy, this work opens a promising pathway toward the realization of energy-efficient, high-density non-volatile memory hybrid architectures.

## Author contributions

Rifat S. Apu developed the methodology, performed the numerical analysis, and drafted the manuscript. M. K. Hasan and A. Marsaad contributed to data analysis. Md H. Rashid and Rubaiyet I. Haque both supervised the study. Rubaiyet I. Haque provided funding and resources and edited the manuscript. All authors reviewed and approved the final manuscript.

## Conflicts of interest

The authors declare that they have no known competing financial interests or personal relationships that could have appeared to influence the work reported in this paper.

## Data Availability

The data and code supporting the findings will be available from the authors on request.
